# Notes on Nursing

**Published:** 1860-11

**Authors:** 


ART. IV.—*Notes on Nursing; what it is, and what it is not.* By FLORENCE NIGHTINGALE. 8vo., pp. 140. New York: D. Appleton & Co., 1860.

AFTER a careful perusal of Miss Florence Nightingale's "Notes on Nursing," we cheerfully commend it to all households as a text-book, and to all mothers and nurses as a guide and companion.

It is a thorough work, almost uselessly minute in its details, one might say, but all the more reliable, as every one knows what an extended field of observation the authoress commanded, and also how great her advantages were for acquiring information during the many years in which she philanthropically devoted herself to tending the sick and wounded. Her experience has been of a wide and diversified nature, and we have a succinct epitome of it in the excellent manual before us. Surely no one could have been better calculated for the composition of such a work than Florence Nightingale, residing, as she did for a long period, in the hospitals of Great Britain, Germany, and Egypt, and finally perfecting and bringing her knowledge to its acme by her sojourn in the Scutari establishments during the Crimean war.

There is probably not a spot in the civilized world where Florence Nightingale's name is not known and recognized as a synonym of all that is self-sacrificing, ennobling, and enduring in woman. Maligned, as she has been, by many who disliked what they termed her *interference* in the Scutari hospitals, and covered with jealous innuendoes, as unworthy as unfounded, this noble woman needs no word in her defense. Her whole life, and the wondrous unselfishness by which it has been characterized throughout, speak "trumpet-tongued" in her praise, and effectually dispel the malicious assertions of her rivals.

In the table of contents prefixed to her "Notes on Nursing," the following subjects are comprised :—

Ventilation and Warming; Health of Houses; Petty Management; Noise; Variety; Taking Food; What Food? Bed and Bedding; Light; Cleanliness of Rooms and Walls; Personal Cleanliness; Chattering Hopes and Advices; Observations on the Sick, etc.

In regard to ventilation, Miss Nightingale tells us that the fear of open windows is a popular fallacy. The sick-room should be thoroughly aired, for with sufficient covering and hot applications a patient can be kept warm in bed while his room is being well ventilated. She writes :—

"You must have open chimneys, open windows or ventilators; no close curtains round your beds; no shutters or curtains to your windows; none of the contrivances by which you undermine your own health or destroy the chances of recovery of the sick.

"The safest atmosphere of all for a patient is a good fire and an open window, excepting in the extremes of temperature."

She mentions one thing that it were well for all to know and remember, namely, that sick persons generally suffer much more from cold in the morning than in the evening. If they are feverish at night, they are almost always chilly ere daybreak. Yet nurses persist in heating the foot-warmer at night, but neglect it in the morning, when it is most needed.

Having been told by one of the best medical authorities that the air in London is never so good as after ten o'clock P.M., Miss Nightingale is a strong advocate for the admission of night air into the sleeping apartments of the sick. Owing to the absence of smoke and noise, she thinks night much the better time for airing the sick-room.

We find the belief in contagion greatly ridiculed in this manual, the authoress thinking that one is much more liable to be attacked by small-pox, measles, or scarlet fever, from living in a dark, ill-aired, unhealthy house, than from constant intercourse with persons suffering from these diseases. She remarks :—

"How few are there who can intelligently trace disease in their households to such causes! Is it not a fact, when small-pox, etc., appears among the children, the very first thought which occurs is 'where' the children can have caught the disease? And the parents immediately run over in their minds all the families with whom they may have been. They never think of looking at home for the source of the mischief. If a neighbor's child is seized with small-pox, the first question asked is, whether it has been vaccinated. No one would undervalue vaccination; but it becomes of doubtful benefit to society when it leads people to look abroad for the source of evils which exist at home.

"We must not forget what, in ordinary language, is called 'infection,' a thing of which people are generally so afraid that they frequently follow the very practice in regard to it which they ought to avoid. Nothing used to be considered so infectious or contagious as small-pox; and people not very long ago covered up patients with very heavy bedclothes, while they kept up large fires and shut the windows. Small-pox, of course, under this regime, is very 'infectious.' People are somewhat wiser now in their management of this disease. They have ventured to cover the patients lightly, and to keep the windows open; and we hear much less of the infection of small-pox than we used to do. But do people in our days act with more wisdom on the subject of infection in fevers, measles, etc., than their forefathers did with small-pox? Does not the popular idea of infection involve that people should take greater care of themselves than of the patient? That, for instance, it is safer not to be too much with the patient, not to attend too much to his wants? Perhaps the best view of duty in attending infectious diseases is afforded by what was very recently the practice, if it is not so even now, in some of the European lazarets, in which the plague-patients used to be condemned to the horrors of filth, overcrowding, and want of ventilation, while the medical attendant was ordered to examine the patient's tongue through an opera-glass, and to toss him a lancet to open his abscesses with.

"True nursing ignores infection, except to prevent it. Cleanliness and fresh air from open windows, with unremitting attention to the patient, are the only defense a true nurse either asks or needs."

Unnecessary noise in the sick-room indicates a thoughtless absence of care, for when a patient is suddenly awakened out of his sleep, the excitement produced is much greater and of a more lasting nature than that which is caused by a continuous sound. Good nursing, however, does not necessarily presuppose an absence of noise; on the contrary, a firm, light step, and a steady, quick hand, are much better than the tip-toe gait and the timid, lingering touch.

Uncertainty and suspense are often more dangerous to the sick than the diseases from which they are suffering, and the delay of a message, or the non-delivery of a letter, have often led to fatal results. Promptness should be considered a *sine qua non* in nursing.

Never hold a whispered conversation about the patient near his room; for, naturally desiring to know the nature of the colloquy, his attention may be strained and his suspicions aroused to feverish and disastrous anxiety.

In feeding a delirious or stupefied person, his attention should first be attracted by the friction of a spoon upon his lips; otherwise, he is liable to suffocation; and the same rule should have a mental application, as speaking suddenly to the sick may produce injurious consequences.

Variety should be an essential element in the sick-room, for none save those who have experienced the feelings can realize what torture it is to lie for weeks in the same room, amid the same surroundings. We should endeavor to make some change rather in the position of light furniture, or by the occasional addition of flowers or paintings.

Miss Nightingale does not admit that cut flowers have any deleterious effects, and says the carbonic acid exhaled from them would not poison a fly.

In impressing upon us the necessity of variety, she writes :—

"Variety of form and brilliancy of color in the objects presented to patients are actual means of recovery.

"But it must be slow variety, *e.g.* if you show a patient ten or twelve engravings successively, ten to one that he does not become cold, or faint, or feverish, or even sick; but hang up opposite him one on each successive day, week, or month, and he will revel in the variety.

"A patient can just as much move his leg when it is fractured, as change his thoughts when no external help from variety is given him. This is, indeed, one of the main sufferings of sickness; just as the fixed posture is one of the main sufferings of the broken limb."

We give the subjoined extracts from the chapter on food :—

"Milk and the preparations from milk are a most important article of food for the sick. Butter is the lightest kind of animal fat, and though it wants the sugar and some of the other elements which there are in milk, yet it is most valuable, both in itself and in enabling the patient to eat more bread. Flour, oats, groats barley, and their kind, are preferable in all their preparations to those of arrow-root, sago, and tapioca. Cream, in many long chronic diseases, is quite irreplaceable by any other article whatever. It seems to act in the same way as beef-tea, and to most it is much easier of digestion than milk. In fact, it seldom disagrees.

"Arrow-root is a great dependence of the nurse. As a vehicle for wine and as a restorative quickly prepared, it is all very well. But it is nothing but starch and water. Flour is both more nutritious and less liable to ferment, and is preferable wherever it can be used.

"Nothing has yet been discovered which is a substitute to the patient for his cup of tea. If you give it at five or six o'clock in the morning, he may perhaps get his only two or three hours sleep during the twenty-four. At the same time, you should never give tea or coffee to the sick, as a rule, after five o'clock in the afternoon. Sleeplessness in the early night is from excitement generally, and is increased by tea or coffee; sleeplessness which continues in the early morning is from exhaustion often, and is relieved by tea. Coffee is a better restorative than tea, but a greater impairer of the digestion.

"An almost universal error among nurses is in the bulk of the food, and especially of the drinks they offer to their patients. Suppose a patient ordered four ounces of brandy during the day, how is he to take this if you make it into four pints with diluting it? The same with tea, arrow-root, milk, etc. You have not increased the nourishment by increasing the bulk; you have very likely diminished both, by giving the patient's digestion more to do, and most probably he will leave half of what he has been ordered to take, because he cannot swallow the bulk with which you have invested it."

It will be unnecessary to refer to the remaining chapters, as they contain but little which is not intuitively known to every good nurse; one thing is mentioned, however, which it would be wrong to overlook; namely, the present lamentable style of woman's attire, and the fact of its utterly incapacitating her for nursing. Indeed, men are now much more efficient in the sick-room. The rustling of dresses, and the many little *gaucheries* consequent upon the wearing of crinoline, are most irritating to the sick.

In nursing, every woman should endeavor to wear garments of the most noiseless material, and should ignore hoops altogether.

In recommending this manual to the public, we would not have it understood that we do so unreservedly, with a full reliance upon its every statement. It undoubtedly contains much which is open to criticism; but as our object has been to give a mere outline of the book, we will leave a critical review of its merits and defects to others.

